# Pycabnn: Efficient and Extensible Software to Construct an Anatomical Basis for a Physiologically Realistic Neural Network Model

**DOI:** 10.3389/fninf.2020.00031

**Published:** 2020-07-07

**Authors:** Ines Wichert, Sanghun Jee, Erik De Schutter, Sungho Hong

**Affiliations:** ^1^Computational Neuroscience Unit, Okinawa Institute of Science and Technology, Onna, Japan; ^2^Bernstein Center for Computational Neuroscience Berlin, Berlin, Germany; ^3^Department of Life Science, Korea University, Seoul, South Korea; ^4^Theoretical Neurobiology, University of Antwerp, Antwerpen, Belgium

**Keywords:** neural network model, anatomical basis, cell position, network connectivity, cerebellum, cerebellar granule cell, Python

## Abstract

Physiologically detailed models of neural networks are an important tool for studying how biophysical mechanisms impact neural information processing. An important, fundamental step in constructing such a model is determining where neurons are placed and how they connect to each other, based on known anatomical properties and constraints given by experimental data. Here we present an open-source software tool, pycabnn, that is dedicated to generating an anatomical model, which serves as the basis of a full network model. In pycabnn, we implemented efficient algorithms for generating physiologically realistic cell positions and for determining connectivity based on extended geometrical structures such as axonal and dendritic morphology. We demonstrate the capabilities and performance of pycabnn by using an example, a network model of the cerebellar granular layer, which requires generating more than half a million cells and computing their mutual connectivity. We show that pycabnn is efficient enough to carry out all the required tasks on a laptop computer within reasonable runtime, although it can also run in a parallel computing environment. Written purely in Python with limited external dependencies, pycabnn is easy to use and extend, and it can be a useful tool for computational neural network studies in the future.

## Introduction

Physiologically realistic neural network simulations are becoming increasingly important in neurobiology studies as they allow investigating experimentally identified biophysical features of a system (Einevoll et al., 2019). However, a large number of network models rely on random anatomical configurations, such as a random positioning of cells in space, and/or random connectivity between them, even when physiological realism is pursued. Those models contradict a growing number of experimental discoveries that reveal non-random anatomical features in diverse neural systems. For example, the locations of cells are not entirely random (Yellott, 1983; Eglen, 2012; Jiao et al., 2014; Maruoka et al., 2017; Töpperwien et al., 2018). The probability of electric and synaptic connections between two cells depends on their mutual distance (Dugué et al., 2009; Rieubland et al., 2014).

Furthermore, recent computational studies demonstrated the functional importance of specific connectivity. In a network, distance limits the spreading of activity from one neuron to others and therefore contributes to localized activity (Pyle and Rosenbaum, 2017; Rosenbaum et al., 2017). Also, if a network has geometric regularity in axonal morphology, patterning of network activity in 3D can emerge (Sudhakar et al., 2017). However, there have not been many easily usable tools that address specifically and systematically the problem of building an anatomical foundation of a neural network model.

In this paper, we present *pycabnn*, a Python tool for Constructing an Anatomical Basis of a Neural Network model. Pycabnn determines positions of cellular structures such as neurons and presynaptic terminals and finds their connectivity, based on experimental measured conditions and physiologically plausible assumptions.

Pycabnn is originally created as a replacement of our previous software, the Boundary Representation Language (BREP), used for constructing a network model of the cerebellar granular layer (Sudhakar et al., 2017). BREP was written in scheme and compiled into a native binary executable by Chicken Scheme^1^ to be deployed in large-scale, multi-cpu computing environments such as cluster supercomputers. In contrast, in designing pycabnn, we emphasized making it portable, expandable, and easy to use. To this end, pycabnn is written purely in the Python language with limited external dependencies on widely used scientific packages, such as *numpy* and *scikit-learn*. It can be used flexibly in diverse computing environments, ranging from a laptop computer to a cluster supercomputer. The Python basis of pycabnn also makes it easy to add enhancements in algorithms and implementations, which significantly improve the performance, compared to BREP. Furthermore, pycabnn specifically aims making a structural basis, and can be used independently of how the network model is finally implemented or which simulation platform is used.

We will explain our core algorithms and how they are implemented in detail in the Methods section. Then, in Results, we will demonstrate a module in pycabnn for generating positions of cells. We will explain the motivation for our core algorithms and experimental backgrounds and show an example of generating positions for various types of cells in a model of the cerebellar granular layer.

We will continue to use the example of the cerebellar granular layer network to demonstrate how another module in pycabnn can be used to find connectivity between neurons. Lastly, we will compare the characteristics and simulation results of a pycabnn-generated model to a similar model that we published in Sudhakar et al. (2017).

## Methods

Pycabnn is an open-source package available at https://github.com/CNS-oist/pycabnn. The documentation for installation and usage can be found in that repository. A list of external packages that pycabnn depends on can be found in “requirements.txt” and “optional-requirements.txt” file in the repository.

### Generation of Cell Positions

For cell bodies and other quasi-spherical cellular structures, we used a stochastic spatial sampling algorithm called Poisson disk sampling (PDS). PDS efficiently generates points covering a space uniformly, or based on a given distribution, with a given minimal distance between them and simulates stochastic dense packing of semi-hard spheres in space. In pycabnn, we implemented a variant of the Bridson algorithm (Bridson, 2007), which uses cubic voxels whose sides are given by $r/\sqrt{D}$ where *r* is the minimal distance between points and *D* is the dimensionality of the space.

Initially, we mark all the voxels “eligible.” Then, we randomly select 20% of the eligible voxels and generate a random point in each selected voxel as cell position candidate. We compute mutual distances of the points and accept only the points satisfying the minimal distance condition (>*r*). The voxels with accepted points are marked “ineligible.” Then, we begin the next round of random voxel selection with the remaining eligible voxels and perform candidate generation. We track a rejection rate of generated candidates to avoid voxels with histories of many rejections. This procedure is repeated until we reach a target number of points or run out of eligible voxels.

We also implemented the maximal PDS algorithm (Ebeida et al., 2012), which generates points until it is impossible to add another point without violating the minimal distance condition. Our implementation of this maximal PDS is mostly the same as the Bridson sampling described above, except for an additional subdivision of voxels step: When the number of newly added points becomes smaller than 0.06% of the target number of points during the Bridson sampling procedure, we split all the eligible voxels (ancestors) into subvoxels (descendants) whose sides are a half of the parent voxel sides. We then remove all the subvoxels that are completely within a minimal distance from any of the previously generated points. Then, random points are generated only within the remaining subvoxels. If a point is accepted, an ancestor voxel containing the point is marked ineligible, just like in the Bridson algorithm, and all of its descendants (subvoxels) are removed. This subdivision step can be performed multiple times, i.e., subvoxels are divided into even smaller subvoxels whenever the number of newly added points becomes too small. As the number of accepted samples increases, the volume that eligible subvoxels can occupy eventually starts to decrease, and this accelerates the sampling procedure.

Our implementation can also generate point clouds that represent multiple cell types mixed together. Rather than spawning them simultaneously, we build one point cloud per cell type sequentially. At each stage, cell positions are generated in the same way with additional rejection rules imposed by previously generated other types of cells.

In some cases, cellular structures are not always distributed isotropically. For example, in the cerebellar cortex, mossy fibers are generally oriented in a sagittal direction, which results in an anisotropic distribution of synaptic terminals (Sultan, 2001). This can be represented by applying a scaling factor to relevant coordinates (see below).

So far, all the algorithms are for packing hard spheres, but cellular structures are soft and allow small deformations for more irregular packing, leading to important differences between the generated and real cellular distribution. For example, in the case of densely packed hard spheres, the number of nearest neighbors would sharply drop as their mutual distance becomes below the minimal distance, while soft spheres would show a more continuous change, just as in experimental data (e.g., Figure 4B). To introduce this additional irregularity, we first subtracted a small *softness margin* from the minimal distance, generated cell positions, and then added small Gaussian noise with standard deviation equal to the softness margin to the generated coordinates. The value of the softness margin can be determined in a several ways. If there is experimental data to compute a histogram of the nearest neighbor distances (e.g., Figure 4B inset), a suitable softness margin can be found by comparing the histograms of the experimental and pycabnn-generated data. A softness margin can also be estimated from cell size variability data. Specifically, for a softness margin δ, we add a random number, drawn from a normal distribution *N*(0, δ^2^), to each cell position coordinate. Therefore, cell size would have a standard deviation of 2δ, which can be compared with the experimental data. In the worst case, without any data, a softness margin can be set by a reasonable assumption about how well a cell body can be compressed.

### Algorithms for Generating Connectivity

#### Overview

Experimental data for electric and synaptic connectivity is often given in the form of a probability density function of the cell-to-cell distance or axon-to-dendrite distance, as described by the so-called Peter’s rule (Rees et al., 2017). In pycabnn, finding connections between spatially extended structures such as axons and dendrites takes the following steps. We first generate the locations of cell somata and then point cloud representations of dendrites and axons by using suitable generators, provided by users. Each point cloud is stored in a data structure, *Query_point*, along with other relevant information per point such as identifiers for the cells and compartments that they belong to. Then, we use an efficient search for the nearest neighbors with the point clouds to identify axodendritic or dendrodendritic connections and also generate necessary information for building a simulation, such as identification of pre-/post-synaptic cells, locations of synapses, axonal propagation delays, etc. Next, we explain how we perform the nearest neighbor search on the point clouds.

#### Algorithm for the Nearest Neighbor Search

In pycabnn, the core algorithm is the K-d tree-based nearest neighbor search, implemented in a Python machine-learning package, *scikit-learn*^2^. In pycabnn, one point cloud representing either the origin or the target structure is organized in the K-d tree whereas the points of the other structure are used one by one as query points.

Briefly, K-d trees are binary search trees that embed points in a *k*-dimensional space. To construct one from a point cloud, we first choose an arbitrary starting point and one of the spatial dimensions. Then, we define a hyperplane that passes through the point and is perpendicular to the chosen axis. This plane separates all points into a left and a right subtree. Next, we choose another axis and perpendicular hyperplane to separate the points in the subtrees again. This step is repeated with circling around the *k* axes until there is only one node left in each subtree, which is then called a leaf node.

When searching for the nearest neighbor of a query point, we first walk down the tree. For each node, we check which side of the hyperplane that goes through the node contains points that are closer to the query point. This procedure is easy since we only have to compare the coordinate of a single axis. The step is performed until we reach a leaf node, which we then register as the “current best.” Then, we walk back up the tree. This time, we check for every node that we visit whether the other side of the hyperplane which goes through the point contains a point that is closer to the query point than the current best. That is the case when the hyperplane intersects with a hypersphere around the query point with a radius of the current best distance. If there is an intersection, we move down to the other subtree. If the leaf node that we find in this process is closer to the query point than the “current best,” we register it as a new “current best.” Then, we again walk up the tree. This iterative process terminates when we reach the root node. In order to find all points within a certain critical radius (ranged search), a similar search is performed, although this time every point that lies within this radius is stored. This algorithm enables a fast search of nearest neighbors, *O*(*kn*log*n*) with *k* being the number of dimensions and *n* being the number of points (Clarkson, 1983).

#### Additional Speedup Implementations

**FIGURE 1:**
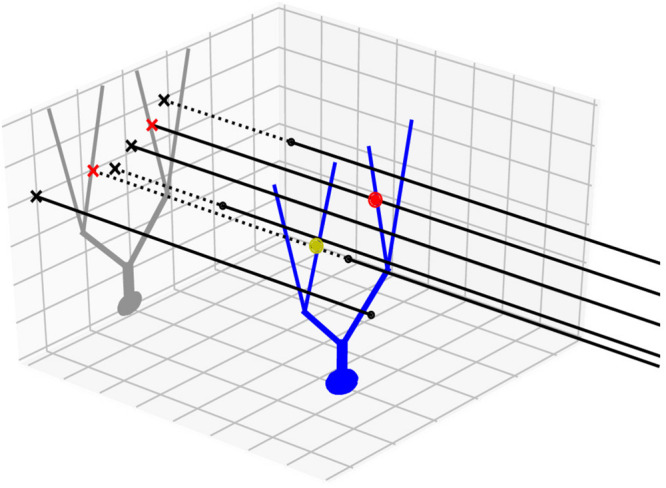
Projection method. An example neuron with an extended morphology (blue) is innervated by multiple axons (black lines), running parallel in 3D. Black dots are axon endpoints and dotted lines are extrapolations of the axons. We project the morphology to a 2D plane (gray), which is orthogonal to the axon direction. The axons are also projected as points (x marks). The axon-cell intersection points are first found in the 2D projection plane (red x’s) and are backtraced to determine whether they correspond to true intersection points in 3D (red dot) or not (yellow dot).

In addition to the nearest neighbor search method in 3D, we also devised and used a 2D projection method, which takes advantage of regularity in the geometry of axon bundles. For example, in the cerebellar cortex, parallel fibers are densely packed, long (a few millimeters) axons that extend along a transverse axis. To represent the fibers in a 3D scheme, we need to generate at least a hundred or more points per axon. However, since they approximately only run in a transverse direction, we can represent them as points projected to a 2D sagittal plane (Figure 1). After projection, a two-dimensional tree can be used to find connections, and as they are now only represented by one point, it is sufficient to only perform one nearest neighbor search per axon. The only additional comparison in the projected axis is necessary to check whether the found points lie within the axonal stretch, making true nearest neighbors in 3D. This method dramatically increases both speed and resource efficiency. Apart from these advantages, this method leads to a more realistic connection density along an axon, since connections are found in a cylindrical region around the axon rather than on the bead-like structure from radii around finite sampled points, which can lead to inaccuracies due to discretization.

To further speed up the process and make effective use of computing power, the search process can be parallelized and performed in cluster supercomputers. We employed a parallel map-and-reduce model of computation and incorporated the package *ipyparallel*^3^, which provides an easy-to-use implementation. In the beginning, we generated point clouds for given cell types, which are packaged in the *Query_point* data structure. Given a source and target point cloud, a K-d tree is constructed from the bigger one at a master computing node, and distributed to multiple worker nodes. Then, we grouped points in the other cloud into chunks, and scattered them to the workers for the parallel nearest neighbor searches. Finally, the results are gathered back to the master node to be saved as files.

### Program Structure

**FIGURE 2:**
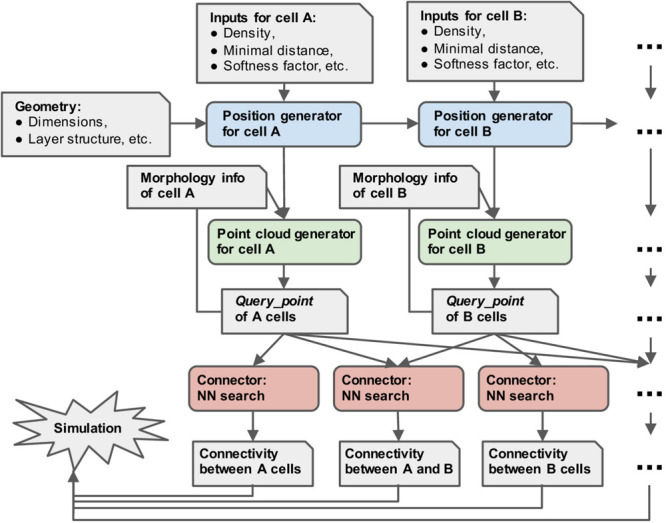
Overview of the structure of pycabnn. After sequentially generating cell body positions of given neuron types (blue), point clouds representing morphological structures are generated (green) and stored in *Query_point* data structures with other information for simulation. Those *Query_point* data are then fed into *Connector’*s (red) that perform the nearest neighbor search and generate connectivity data in a format that a simulator can take as an input.

Figure 2 shows the workflow with pycabnn. We begin with a specification of the model such as the model size, etc. Then, the positions of cells belonging to each type are generated sequentially, and *Cell_pop* objects are created based on them. After that, for each cell type, we render a point cloud by calling a rendering method of the *Cell_pop* module, which is stored in a *Query_point* object with relevant information, such as the IDs of enclosing segments. When the *Query_point*s are ready, we use the *Connector* objects to generate connectivity from one cell type to another. The last step outputs a list of (source cell ID, target cell ID, target segment ID, distance, etc.) and stores it in one or multiple files.

### Data Generation and Simulation Procedure

For an example of cell position generation in section “Generation of Cell Positions,” we created a position model of the granular layer, with a size of 700 μm (mediolateral) × 700 μm (sagittal) × 200 μm (vertical) in the rodent cerebellum. We sequentially generated Golgi cells (GoC), glomeruli (Glo), and granule cells (GC). We chose this order since we considered that generating the GC positions lastly would be better for replicating the dense volume-filling by GCs seen in experimental data (Töpperwien et al., 2018).

For each cell type, we first computed the target number of cells, *n*_*cell*_, from experimental data of the cell densities (see section “Results”). When the target volume is *V*, the predicted minimal distance based on the complete filling assumption, *d*_*c*_, is

$$V=n_{c\,e\,l\,l}\cdot \frac{4}{3}\,\pi \,{\left(\frac{d_{c}}{2}\right)}^{3}⟹d_{c}={\left(\frac{6\,V}{\pi \,n_{c\,e\,l\,l}}\right)}^{1/3}.$$

Initially, we set the first minimal distance to test, *d*_1_, to a value slightly larger than *d*_*c*_. Then, we iteratively searched for a threshold value, *d*_θ_, where a slight increase causes the number of generated cells to become smaller than *n*_*cell*_. We also considered the anisotropy in the Glo distribution such that the Glo-to-Glo distance is about three times larger in the mediolateral than parasagittal direction (Sultan, 2001), and used a squeezed coordinate system for Glos (see section “Generation of Cell Positions” for details of this procedure). Since our algorithms are stochastic, the same minimal distance parameter can lead to cell distributions with a different density. To check this, we ran the maximal PDS algorithm with *d*_θ_ to generate as many cells as possible and monitored whether the deviation in their density from the target is small (<0.01%).

Furthermore, to avoid boundary effects, we generated cells in an extended volume that is 50 μm larger in every axis than the target volume and removed all the cells contained in 25 μm-wide strips at boundaries. Then, all the coordinates are shifted by 25 μm so that the lowest corner of the volume becomes (*x*, *y*, *z*) = (0, 0, 0) again. All the cell positions are generated with a MacBook Pro (2.9 GHz Intel Core i7 with 16 GB RAM; Apple Inc., CA, United States) in single-core mode.

In section “Generation of Connectivity,” we generated the connectivity for a published network model (Sudhakar et al., 2017). For a better comparison of results, we used the same cellular positions as one of the simulations in the study, instead of the cell position generator of pycabnn. Then, we ran BREP, the software used in Sudhakar et al. (2017), and pycabnn with the same cell position data, in the OIST *sango* cluster supercomputer with 120 cores (Intel Xeon E5-2680v3, 5 GB RAM per cpu). We ran the performance tests of pycabnn with the same setup as mentioned above.

Network simulations, based on the connections by BREP or pycabnn, ran in the *sango* cluster computer with 200 cores (see above). We used the exactly the same model code^4^ built on the NEURON 7.4 simulation platform and used identical parameters to those reported in Sudhakar et al. (2017) other than the connectivity. The simulation paradigm was the same as for Figures 2E–H in Sudhakar et al. (2017) (with gap junctions) where a small number of mossy fibers fired at 60 Hz, beginning from *t* = 500 ms.

## Results

Here, we show an example of how pycabnn is used and demonstrate its performance with a network model for the granular layer of the rodent cerebellar cortex. Briefly, this model is composed of two cell types, excitatory granular cells (GC) and inhibitory Golgi cells (GoC). Both types of neurons receive external inputs from mossy fibers (MF) that enter from the bottom of the granular layer, and branch to form glomeruli (Glo), distinctive intertwinings between MF presynaptic terminals, GC dendrites, and GoC axons. Each GC emits a long axon that initially ascends in a vertical direction, and bifurcates in another region called the molecular layer, to travel extensively in a transverse/mediolateral direction. GoCs receive excitatory inputs both from the ascending segment (ascending axon; AA) and the transversely stretched part (parallel fiber; PF), in addition to the MF input.

### Generation of Cell Positions

**FIGURE 3:**
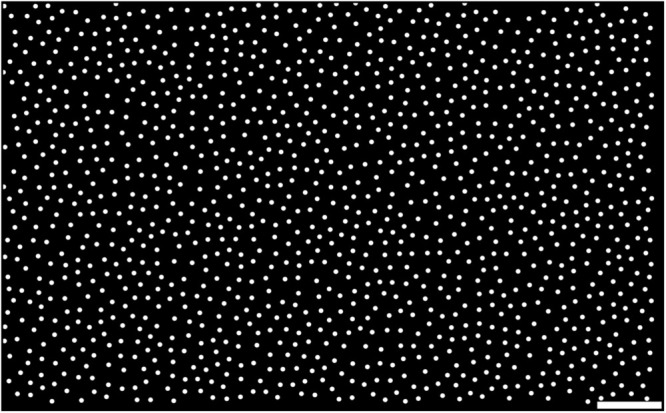
Example of generated points by the Poisson disk sampling method. Samples are generated by the maximal PDS algorithm in 2D. The density and mutual spacing were given by ρ_MF_ = 1650 mm^– 2^ (Sudhakar et al., 2017) and *r*_MF_ = 20.9 μm, respectively, while each point is plotted with a diameter of 8 μm for visual clarity. Scale bar: 100 μm.

In many neural systems, positions of the cells are not entirely random but often follow patterns similar to densely packed soft granules, such as retinal cells (Yellott, 1983; Eglen, 2012; Jiao et al., 2014; Maruoka et al., 2017; Töpperwien et al., 2018; Yellott, 1983; Jiao et al., 2014), cortical neurons in microcolumns (Maruoka et al., 2017), etc. Cerebellar GCs also show a large peak in their pair correlation function, and therefore the distance to the nearest neighbor is typically around the average diameter of GCs, suggesting that they are densely packed (Töpperwien et al., 2018). The cell position generator of pycabnn employs an efficient algorithm, called the Poisson disk sampling (PDS), for generating points in a densely packed system, with a capability to generate heterogeneous cell populations (see section “Methods”). In particular, we implemented the maximal PDS algorithm by Ebeida et al. (2012) that finds a maximal filling of a volume by balls, in which an additional ball cannot be inserted without violating a minimal distance condition. For example, Figure 3 shows uniformly distributed points in 2D, generated by the algorithm, as the positions of mossy fibers (MF) entering the cerebellar granular layer, with a density ρ_MF_ = 1650 mm^–2^ (Sudhakar et al., 2017) and mutual spacing *r*_MF_ = 20.9 μm.

By using the maximal PDS method in pycabnn, we sequentially generated GoCs, Glos, and GCs in a volume of 700 μm (mediolateral) × 700 μm (sagittal) × 200 μm (vertical), with densities ρ_GoC_ = 9500 mm^–3^ (Dugué et al., 2009) and ρ_GC_ = 1.9 × 10^6^ mm^–3^ (Billings et al., 2014). For Glos, we used that each GC makes synapses to 4.5 glomeruli on average and also each glomerulus receives 15 GC dendrites on average (Palay and Chan-Palay, 1974), which leads to a number of glomeruli per GC = 4.5/15 = 0.3. Therefore, we used ρ_Glo_ = 0.57 × 10^6^ mm^–3^.

For each cell type in this model, we determined a minimal mutual distance between cells that can achieve maximal filling of the volume with a given density, e.g., the filling where we cannot add one more cell without violating the minimal mutual distance. We found that a minimal mutual distance for GoCs was *r*_GoC_ = 45 μm, with a softness margin (see section “Methods”) of 1 μm, which we set by the assumption that a GoC somata is as soft as glomeruli (see below). Note that *r*_GoC_ is well above the diameter of GoCs, *d*_GoC_ = 27 μm (Solinas et al., 2007).

Glomeruli are known to occupy about one-third of the whole volume (Billings et al., 2014) and are also anisotropically distributed as their mutual distance tends to be about three times larger in sagittal than in mediolateral direction (Sultan, 2001). To satisfy these two conditions, we generated glomeruli in a virtual, squeezed volume, which is created by shrinking the sagittal axis of the original volume by 1/3 and tuned the minimal distance *r*_Glo_ in this squeezed volume. The glomerulus density ρ_Glo_ estimated above predicts *r*_glo_ ≈ 7–9 μm, which is close to experimentally measured sizes of glomeruli in rats [8–12 and 6–9 μm along the longer and shorter axis, respectively (Jakab and Hámori, 1988)]. Therefore, we assumed that the minimal mutual distance between glomeruli is given by a glomerulus diameter, *d*_Glo_ = *r*_Glo_. This diameter was also used to determine how GoCs and glomeruli avoid each other. We estimated that a softness margin of 1 μm approximately corresponds to the size variability of glomeruli above (Jakab and Hámori, 1988). With this softness margin, we found that, if *r*_glo_ = *d*_Glo_ = 8.39 μm, glomeruli filled the given squeezed volume with ρ_Glo_ with approximate maximality. Lastly, we performed a similar procedure for GCs and obtained the minimal distance/diameter of a GC, *r*_GC_ = *d*_GC_ = 6.15 μm and a softness margin of 0.2 μm, which we estimated by comparing the nearest neighbor density histogram with that from the experimental data (see section “Methods” and below). This is close to but slightly smaller than an experimental measurement *d*_GC_ = 6.7 μm (Billings et al., 2014). This can be caused by our model GCs being semi-hard spheres, while real GCs and glomeruli are soft structures. Therefore, they can have larger average sizes than our models but can be squeezed better to fill the same volume.

**FIGURE 4:**
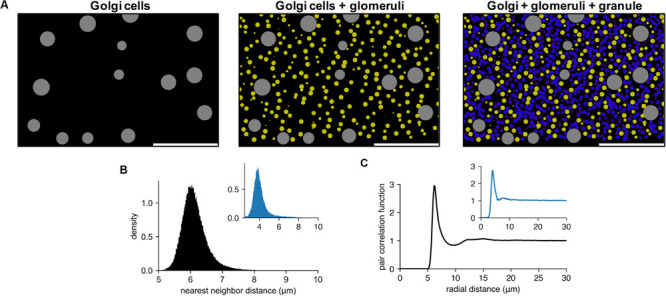
Generated cell locations in the cerebellar granular layer. **(A)** Sequential generation of Golgi cells (Left), glomeruli (Middle), and granule cells (Right) in a model of the granular layer in the rodent cerebellum. A small fraction of the cells is shown in a sagittal plane (*x*: sagittal, *y*: vertical direction). Scale bar: 100 μm. Physiological diameters (Golgi: 27 μm; Glomerulus: 8.39 μm; Granule: 6.15 μm) and density parameters (Billings et al., 2014; Sudhakar et al., 2017) are used. **(B,C)**. Nearest neighbor distribution **(B)** and pair correlation function **(C)** of the model granule cells. Insets are based on experimental data from human cerebellum (Töpperwien et al., 2018). Peak locations are different in insets since they are based on human data where the granule cells are smaller ([diameter: 4.00 ± 0.02 μm (Töpperwien et al., 2018)].

Figure 4A shows an example of generated cell populations, demonstrating that pycabnn produced the cell locations without any significant overlap within given minimal distances and/or cell body sizes. The quantitative analysis further confirmed this. Particularly for GCs, we computed a histogram of distances between two nearest neighboring cells (Figure 4B) and this showed a peak at an average cell diameter *d*_GC_ = 6.15 μm that sharply declines as distance decreases. This proves that neighboring GCs are never significantly closer to each other than a GC diameter and are most likely touching each other, as in tight packing. The pair correlation function, computed by averaging the density functions around each GC, shows similar properties (Figure 4C). Around each GC, there is no cell within 5 μm and the density of other GCs again peaks at *d*_GC_ = 6.15 μm. Notably, there is no significant secondary or further peak beyond that point, which would be present if GCs formed a lattice-like structure. These two features in the generated GC positions are qualitatively congruent with experimental data from human cerebellum (Töpperwien et al., 2018; Figures 4B,C insets), which proves that our algorithm indeed produces realistic cell positions.

### Generation of Connectivity

#### Finding Connectivity by Cell-to-Cell Distance

When experimental data for connectivity is given by the probability to connect or connection strength with respect to cell body-to-cell body distance, generating connections based on cell locations is comparatively easy.

Connections from glomeruli to GCs were generated by a simple distance-based search scheme as described in Sudhakar et al. (2017), which finds possible connections to distinct neighboring glomeruli within a certain range from each GC, rather than fixing the number of connections per GC. Here, we additionally considered that GCs prefer making connections to glomeruli in a sagittal direction and therefore their dendrites are stretched about four times longer in a sagittal than mediolateral direction (Houston et al., 2017). We incorporated this by performing our ranged search in a coordinate system that is squeezed by 1/4 in sagittal direction, similar to the coordinate scaling that we used for generating glomeruli positions.

With this procedure and the cell positions determined as described in the previous section, we got realistic 4.43 ± 1.37 connections per GC when the search range in mediolateral direction was *r*_*GC–Glo*_ = 7.85 μm. In this case, the GC-to-Glo distance, or length of our model GC dendrite was 13.47 ± 5.81 μm, which is close to reported estimates from experimental data (Hámori and Somogyi, 1983; Billings et al., 2014).

Notably, when the cell positions were purely random, we found 4.25 ± 2.12 connections per GC, and therefore a variability in GC-Glo connections increased by 56% compared to our maximal volume-filling model. Thus, there are far less outlier GCs in the volume-filling model. For example, in the random position model, GCs with more than seven or less than three connections were 7 and 21% of all GCs, respectively. On the other hand, in the volume-filling model, those GCs were only 1 and 7%, respectively. This shows that the volume-filling model provides a natural uniformization mechanism for the number of GC inputs. Similar input uniformization in densely packed neurons has also been reported previously (Yellott, 1983; Jiao et al., 2014). Our cell position and connectivity model suggests that dense packing of neurons, which explains a comparatively small volume of the cerebellum (Töpperwien et al., 2018), is related to similar functional advantages, such as improved sampling of inputs (Yellott, 1983) by GCs.

#### Finding Connectivity by Tracking Axons

Establishing connectivity becomes more complicated when we need to consider extended axonal and dendritic geometry. In our example of the cerebellar granular layer, GC axons have a unique geometric feature that makes it inadequate to determine GC-to-GoC connectivity by simple distance-based rules. GC axons vertically rise from a cell body in the granular layer to branching points in the molecular layer, forming ascending axons (AA). Then, they bifurcate and elongate parallelly in a transverse direction for a few millimeters, called parallel fibers (PF). Both parts have presynaptic terminals and can make excitatory synaptic connections with other types of neurons. To fully account for this geometric configuration, it is necessary to virtually construct GC axons and track them to find potential synaptic connections with GoCs.

**FIGURE 5:**
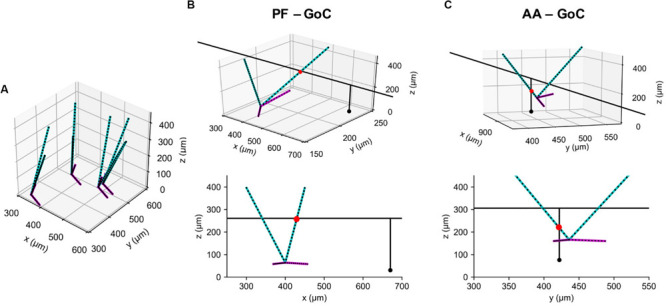
Example synaptic connections of GoC and GC axons. **(A)** Model GoCs (cyan: apical, magenta: basal dendrites) representing their extended morphologies. Black dots are sampling points in point clouds. Only four GoCs are shown for clarity. **(B)** An example synaptic connection (red dot) between a GoC and PF segment of GC axon (black line), shown in 3D (Top) and in an *x-z* plane (Bottom). A black dot represents a GC emitting the axon. **(C)** The same figure as **(B)** for an AA-GoC connection example. *x*: mediolateral, *y*: parasagittal, *z*: vertical axis.

As in our previous model (Sudhakar et al., 2017), we used a GoC model with two apical and two basal dendrites, both oriented randomly with a preference toward the sagittal direction. Again, as in this model, we represented them by point clouds, each containing 25 and 12 points sampled uniformly along each of the apical and basal dendrites, respectively (Figure 5A). Note that each dendrite is segmented via the lambda rule (Hines and Carnevale, 1997) and all the points carry information about to which dendrites and segments they belong. This information is stored in a *Query_point* structure with the positions. Due to their lengths, each GC axon needs to be represented by more than at least a few hundred points. We took advantage of their geometric properties that AAs and PFs run in parallel in a vertical and transverse direction, respectively, and used a 2D projection scheme. It represents each structure as its intersection point with the plane perpendicular to it, together with endpoint coordinates in the projected direction (Figure 1; see section “Methods”).

With generated point clouds for the two populations, pycabnn correctly identified candidate synaptic connections both for AAs and PFs (Figures 5B,C). Compared to our previously used software BREP, pycabnn showed much superior performance. In parallel runs on a cluster computer with 120 cores, pycabnn ran 2.6 times faster (pycabnn: 333.31 ± 3.8 s, averaged over 5 runs; BREP: 864.24 ± 38.27 s, averaged over 17 runs), given the same initial cell positions. The speedup in the cluster computer could have been limited by communication cost between nodes. Indeed, on a laptop computer in single-core mode, the runtime of pycabnn was comparable to that of BREP in the cluster computing environment (829.21 ± 37.30 s, averaged over 5 runs).

**FIGURE 6:**
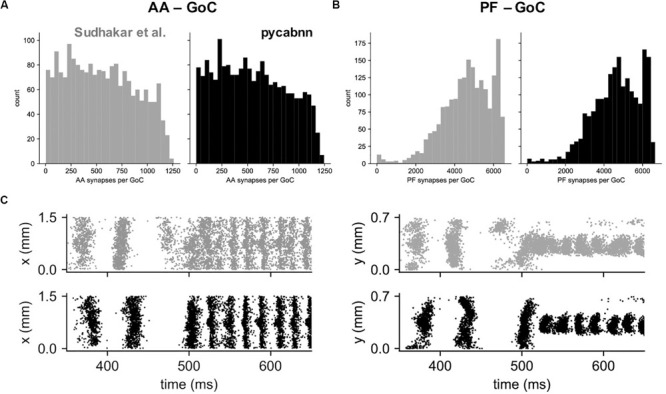
The network model constructed by pycabnn replicates connectivity and behavior. **(A)** The number of synaptic connections between ascending axons (AA) and GoCs in the Sudhakar et al. model (left, gray) and pycabnn (right, black). **(B)** The same figure as A, for parallel fiber axons (PF). **(C)** Firing of GoCs in the Sudhakar et al. model (top, gray) and network constructed by pycabnn (bottom, black). The same data is plotted in time vs. GoC *x* positions (left) and also *y* positions (right). *x*: mediolateral, *y*: parasagittal, *z*: vertical axis.

Other than performance differences, we found that BREP and pycabnn generated similar connectivity structures for the network model. For example, the number of synaptic connections per GoC shows a very similar distribution, both for the AA and PF case (Figures 6A,B). When we ran the network model simulation in Sudhakar et al. (2017); see section “Methods” based on the pycabnn-generated connectivity, the result was also comparable to the BREP-based model. It showed, for example, the characteristic oscillation of GoC firing, dependent on the mossy fiber firing rate (Figure 6C).

In summary, this result demonstrates that the dendritic/axonal morphology generation and connectivity determination of pycabnn produces correct, expected results, given the specifications of a network model, and with high efficiency.

## Discussion

We introduce pycabnn, a software tool to construct an anatomical basis for a physiological, large-scale neural network simulation. Pycabnn is built with efficient algorithms for two stages of building a model, generating positions of neurons and determining their mutual connectivity. In the first part, we implemented efficient algorithms for stochastic packing of spheres that can be used for making a volume-filling model of cell distribution. In the second part, pycabnn uses a fast search algorithm for nearest neighbors using a K-d tree method, to determine connectivity based on the distance between cellular structures.

We tested pycabnn with a physiologically detailed model of the cerebellar granular layer. We found that pycabnn can generate cell positions that are congruent with recent experimental data. As for the connectivity part, we confirmed that pycabnn generates connectivity that is closely compatible with an existing network model and simulation, showing very similar activity patterns to the previous model built with our previous software, BREP. BREP was written in scheme and compiled to a native binary program to run on distributed multiple CPUs. However, the connectivity generation by pycabnn was more efficient than by BREP, though pycabnn was written Python, an interpreted scripting language. Furthermore, the same model can be built on a single laptop computer with a reasonable runtime.

The cell placement algorithm of pycabnn is suited for building a maximal volume-filling model of cell distribution. This is inspired by the experimental findings of quasi-random neuronal distributions in diverse neural systems. In particular, our major target system, the cerebellar cortex, has been shown to have such features, which were replicated by our pycabnn model. A maximal volume-filling model predicted the sizes of glomeruli and GCs given their densities based on an assumption that the volume is maximally occupied. Those sizes were close to experimental measurements, confirming that indeed the cerebellar granular layer is a densely packed system (Töpperwien et al., 2018). The maximal volume-filling model had implications for synaptic connectivity, primarily reducing the variability in GC-to-glomerulus connections formed by a distance-based search. It has been noted that a quasi-random cell distribution is advantageous since each neuron can sample its inputs with enhanced uniformity (Yellott, 1983; Jiao et al., 2014). This illustrates the importance of having a good cell placement model for building a network model, which was our original motivation for implementing this step in pycabnn.

In particular, the average number of GC-to-glomerulus connection per GC has been repeatedly related to optimal input/output information transfer by GCs (Marr, 1969; Billings et al., 2014; Litwin-Kumar et al., 2017). However, the variability of connection and how it impacts information processing have been rarely studied. Our realistic position model of both GCs and glomeruli suggests that the comparatively small volume of the cerebellum resulting in dense packing of the cells contributes to reducing the connection variability and improves the information transfer by GCs, for example by uniformizing the dimensionality expansion of the mossy fiber inputs (Litwin-Kumar et al., 2017). However, a quantitative study on the impact of the connection variability is much beyond the scope of this paper, and we will leave it to future study.

When determining network connectivity, pycabnn assumes that experimental data constraining the connections are given by the so-called Peter’s rule (Rees et al., 2017), predicting synapses based on the distance between cells or extended cellular structures such as dendrites and axons. Implementing this can sound straightforward, but several optimizations were made to achieve sufficient efficiency in pycabnn. For example, in the nearest neighbor search, due to the difference in complexity of building a K-d tree and performing a single search, it is generally faster to make a tree with a bigger point cloud if searches are performed between two unequal-sized clouds of a source and target. Therefore, in our implementation of the nearest neighbor search, a K-d tree is always made from a bigger point cloud and search results are adaptively repackaged depending on whether the tree is built from a source or target. Also, we implemented a 2D projection scheme (Figure 1) that takes advantage of geometric regularity in axonal and dendritic morphology. This is particularly useful in our main application, the cerebellar cortex, where parallel fibers and ascending axons from GCs run parallel to each in one direction but can be potentially useful in other systems with similar geometrical properties. Compared to our previous software, BREP, which also used the K-d tree for connectivity determination, pycabnn ran much faster due to these implementation details designed for efficiency.

By using the Peter’s rule, pycabnn inevitably shares its issues. First, Peter’s rule just describes the proportion of synapses and therefore it may not describe the general network structure. Second, it is not enough to make synapses just based on distance between axons and dendrites, since there can be several factors affecting the formation of synapses, such as neurogeometry (location, orientation and branch morphometrics) and other synaptic features including spines, shafts, gap junctions, and terminal boutons. Last, depending on at which level Peter’s rule would be applied, different results will be obtained (Rees et al., 2017).

Pycabnn has other limitations, and we will make improvements in those aspects in the future. First of all, cell position generation is limited to a model of volume filling by hard spheres. Our approach is comparable to a recent study by Casali et al. (2019), who used a different method, the Bounded Self-Avoiding Random Walk Algorithm (BSRW), which also implements cell placement without overlapping between cells within critical ranges. However, in comparisons with the experimental data, which they did not do, the experimental data (Figures 4B,C insets) clearly shows that the minimal distance distribution is rather smooth without a discontinuity, which is the feature that a model with packed hard spheres cannot achieve. We tried to circumvent this difficulty by including random perturbation set by a softness margin parameter. In comparison, other studies explicitly modeled the packing of soft discs in two dimensions and yielded very realistic cell distributions (Eglen et al., 2000; Jiao et al., 2014). However, it is also necessary to evaluate the cell-to-cell interactions in those schemes, which can be computationally expensive. There are known efficient algorithms for simulating many-body interactions, e.g., the Barnes-Hut method (Barnes and Hut, 1986) that can be adapted for our purpose.

Another limitation is that pycabnn can generate duplicate connections: If cell structures are close to each other within a critical range over a long enough distance, pycabnn will find a cluster of multiple adjacent connections in a small region. Although multiple, clustered connections between an axon and dendrite have been observed in real systems (Bloss et al., 2018), many of them can arise as artifacts in the construction of a model. A simple, possible solution is to restrict the number of connections made between any two structures, which can be done in a post-processing step. Other promising potential improvements include handling of detailed morphology of neurons via point cloud generation from a reconstructed morphology and a scheme to divide a volume and process subvolumes in parallel, and merge results for further scaling-up.

Pycabnn is an open-source program written almost purely in Python 3, with dependency only on a few widely used open-source science packages, which makes using and extending pycabnn easy. Pycabnn output, containing the structural basis, can be used with most of simulation platforms. Since large-scale imaging data of cell positions and connectivity are increasingly available, we believe pycabnn, as a tool to model those aspects, will be useful for computational neural network studies in the future.

## Data Availability Statement

The datasets generated for this study, together with the codes, can be found in https://github.com/CNS-OIST/pycabnn.

## Author Contributions

SH and ED conceived the research. IW and SJ wrote an initial version of the software. IW, SJ, and SH tested and revised the software. All authors wrote the manuscript and approved the submitted version.

## Conflict of Interest

The authors declare that the research was conducted in the absence of any commercial or financial relationships that could be construed as a potential conflict of interest.

## Footnotes

1 https://www.call-cc.org

2 https://scikit-learn.org

3 https://pypi.org/project/ipyparallel

4 https://senselab.med.yale.edu/ModelDB/ShowModel?model=232023
